# The Change of a Medically Important Genus: Worldwide Occurrence of Genetically Diverse Novel *Brucella* Species in Exotic Frogs

**DOI:** 10.1371/journal.pone.0168872

**Published:** 2016-12-30

**Authors:** Holger C. Scholz, Kristin Mühldorfer, Cathy Shilton, Suresh Benedict, Adrian M. Whatmore, Jochen Blom, Tobias Eisenberg

**Affiliations:** 1 Bundeswehr Institute of Microbiology, Neuherbergstrasse 11, Munich and German Center for Infection Research (DZIF), Munich, Germany; 2 Department of Wildlife Diseases, Leibniz Institute for Zoo and Wildlife Research, Germany; 3 Berrimah Veterinary Laboratories, Northern Territory Government, Berrimah, Northern Territory, Australia; 4 Animal & Plant Health Agency, Woodham Lane, Addlestone, United Kingdom; 5 Center for Biotechnology, CeBiTec, Universität Bielefeld, Bielefeld, Germany; 6 Hessian State Laboratory (LHL), Schubertstrasse 60, Giessen, Germany; East Carolina University Brody School of Medicine, UNITED STATES

## Abstract

The genus *Brucella* comprises various species of both veterinary and human medical importance. All species are genetically highly related to each other, sharing intra-species average nucleotide identities (ANI) of > 99%. Infections occur among various warm-blooded animal species, marine mammals, and humans. Until recently, amphibians had not been recognized as a host for *Brucella*. In this study, however, we show that novel *Brucella* species are distributed among exotic frogs worldwide. Comparative *recA* gene analysis of 36 frog isolates from various continents and different frog species revealed an unexpected high genetic diversity, not observed among classical *Brucella* species. In phylogenetic reconstructions the isolates consequently formed various clusters and grouped together with atypical more distantly related brucellae, like *B*. *inopinata*, strain BO2, and Australian isolates from rodents, some of which were isolated as human pathogens. Of one frog isolate (10RB9215) the genome sequence was determined. Comparative genome analysis of this isolate and the classical *Brucella* species revealed additional genetic material, absent from classical *Brucella* species but present in *Ochrobactrum*, the closest genetic neighbor of *Brucella*, and in other soil associated genera of the *Alphaproteobacteria*. The presence of gene clusters encoding for additional metabolic functions, flanked by tRNAs and mobile genetic elements, as well as by bacteriophages is suggestive for a different ecology compared to classical *Brucella* species. Furthermore it suggests that amphibian isolates may represent a link between free living soil saprophytes and the pathogenic *Brucella* with a preferred intracellular habitat. We therefore assume that brucellae from frogs have a reservoir in soil and, in contrast to classical brucellae, undergo extensive horizontal gene transfer.

## Introduction

Brucellosis, caused by Brucella spp., is a zoonosis and a disease of both veterinary and public health significance worldwide. The majority of human infections are acquired through transmission from infected animals, either by direct contact or by the consumption of contaminated food products like meat of milk.

The genus comprises several important highly pathogenic species and can be divided into the classical brucellae (*B*. *melitensis*, *B*. *abortus*, *B*. *suis*, *B*. *canis*, *B*. *ovis*, *B*. *neotomae*), brucellae infecting marine mammals (*B*. *ceti* and *B*. *pinnipedialis*), and the more recently identified species (*B*. *microti*, *B*. *inopinata*, *B*. *papionis*, and *B*. *vulpis*). Additional strains from various human and animal sources are currently awaiting final genus affiliation. *Brucella microti*, *B*. *inopinata*, and *B*. *vulpis* belong to the ‘atypical’ group of brucellae, exhibiting either atypical phenotypic traits (*B*. *microti*) or represent genetically more distantly related species (*B*. *inopinata* and *B*. *vulpis*).

Human infections are mainly caused by classical *Brucella* species, in particular *B*. *melitensis*, which contributes to 98% of all human brucellosis cases. Within the ‘atypical’ brucellae, only *B*. *inopinata* and the non-classified *Brucella* strain BO2 were isolated from human infections [1, 2]. Despite a pronounced natural host preference, *Brucella* species can infect various warm-blooded animal species. *Brucella ceti* and *B*. *pinnipedialis* are known to infect marine mammals but human infections caused by these two species are very rare [3]. The natural hosts of *B*. *inopinata* and strain BO2 are still unknown.

All classical *Brucella* species are genetically highly related to each other with average core genome nucleotide identities (ANI) of >99% and harbor identical 16S rRNA and *recA* gene sequences [4]. ‘Atypical’ *Brucella* species like *B*. *inopinata* and *B*. *vulpis* still have ANI values of 98% compared with the type species, *B*. *melitensis*, but show a few differences (2–5 nucleotides) in their 16S rRNA and *recA* gene sequences [5, 6]. The genomes of some of the ‘atypical’ *Brucella* species (*B*. *inopinata*, *B*. *vulpis*, strain BO2) carry additional genetic material not found in classical *Brucella* species but present in other soil associated bacteria of the *Alphaproteobacteria*. Most of the accessory genes encode for additional metabolic functions or represent bacteriophages and mobile genetic elements, which indicates a different ecology in comparison to the classical host-adapted *Brucella* species.

Within the last three years, novel ‘atypical’ brucellae emerged from exotic frogs, a host not previously recognized as a reservoir for *Brucella*. Notably this concerns various frog species from different continents, including Africa, South and Central America, Asia, and Australia. To date, four cases of *Brucella* infections in frogs have been published. The first report describes the isolation of *Brucella* sp. from wild-caught African bullfrogs (*Pyxicephalus edulis*) imported from Tanzania in a quarantine centre of a zoo in Germany [7]. The bullfrogs displayed localized or systemic granulomatous lesions that could not reliably be distinguished from co-occurring mycobacterial and/or fungal infections (T. Eisenberg, unpublished). The second publication reports the isolation of a *Brucella inopinata*-like strain from subcutaneous abscess material of a big-eyed tree frog (*Leptopelis vermiculatus*) bought from a pet shop in Germany [8], whilst the third case [9] was reported from the UK in a White’s tree frog (*Litoria caerulea*) with fluid-filled skin lesions. The most recent case of *Brucella* infection was described in a Pac-Man frog (*Ceratophyrus ornate*) at a veterinary hospital in Texas; USA [10].

Meanwhile ‘atypical’ brucellae were also identified in tomato frogs (*Dyscophus antongilii*), a red-eyed tree frog (*Agalychnis callidryas*) and Amazonian milk frogs (*Trachycephalus resinifictrix*) from two zoos in Germany (this study) as well as in cane toads (*Chaunus* [*Bufo*] *marinus*) from Australia [11] (this study).

In order to determine the genetic diversity among brucellae isolated from frogs we characterized 36 ‘atypical’ *Brucella* strains, isolated from various frog species of different geographical origins by *recA* gene sequence analysis. In the case of one strain the whole genome sequence was determined and compared to classical *Brucella* species.

## Material and Methods

### *Brucella* isolates and frog species

A total of 36 atypical Brucella isolates were investigated. The majority of the isolates were obtained from African bullfrogs (*P*. *edulis*) imported from Tanzania and kept in quarantine in a zoo in Germany in 2009 [7]. Details on all *Brucella* strains including host species with geographical origin and cross-pathology, and country and year of isolation are provided in Table 1. Seven isolates from Australian cane toads were originally identified as *Ochrobactrum anthropi* [11] and could be reclassified as *Brucella* sp. in this study.

**Table 1:** History and origin of ‘atypical’ *Brucella* strains isolated from amphibian host species. (wc): wild caught, (cb): captive bred.

Strain designation	Host species	Geographical origin	Gross pathology	Country, year of isolation	Reference
07 0194 A	Cane toad^1^ (wc)	Tropical Americas, invasive species in Australia	spinal arthropathy	AUS, 2008	(11), this study
07 0064 B	Cane toad^1^ (wc)	Tropical Americas, invasive species in Australia	spinal arthropathy	AUS, 2008	(11), this study
07 0064 C	Cane toad^1^ (wc)	Tropical Americas, invasive species in Australia	spinal arthropathy	AUS, 2008	(11), this study
07 0194 C	Cane toad^1^ (wc)	Tropical Americas, invasive species in Australia	spinal arthropathy	AUS, 2008	(11), this study
07 0064 E	Cane toad^1^ (wc)	Tropical Americas, invasive species in Australia	spinal arthropathy	AUS, 2008	(11), this study
07 0194 E	Cane toad^1^ (wc)	Tropical Americas, invasive species in Australia	spinal arthropathy	AUS, 2008	(11), this study
09RB8471	African bull frog^2^ (wc)	Southeast, Central and Western Africa	granulomatous / purulent dermatitis	GER, 2009*^A^	(7)
09RB8908	African bull frog^2^ (wc)	Southeast, Central and Western Africa	granulomatous / purulent dermatitis	GER, 2009*^A^	this study
09RB8909	African bull frog^2^ (wc)	Southeast, Central and Western Africa	granulomatous / purulent dermatitis	GER, 2009*^A^	this study
09RB8910	African bull frog^2^ (wc)	Southeast, Central and Western Africa	granulomatous / purulent dermatitis	GER, 2009*^A^	this study
09RB8913	African bull frog^2^ (wc)	Southeast, Central and Western Africa	granulomatous / purulent dermatitis	GER, 2009*^A^	this study
09RB8914	African bull frog^2^ (wc)	Southeast, Central and Western Africa	granulomatous / purulent dermatitis	GER, 2009*^A^	this study
09RB8915	African bull frog^2^ (wc)	Southeast, Central and Western Africa	granulomatous / purulent dermatitis	GER, 2009*^A^	this study
09RB8918	African bull frog^2^ (wc)	Southeast, Central and Western Africa	granulomatous / purulent dermatitis	GER, 2009*^A^	this study
10RB9205	African bull frog^2^ (wc)	Southeast, Central and Western Africa	granulomatous / purulent dermatitis	GER, 2009*^A^	this study
10RB9206	African bull frog^2^ (wc)	Southeast, Central and Western Africa	granulomatous / purulent dermatitis	GER, 2009*^A^	this study
10RB9207	African bull frog^2^ (wc)	Southeast, Central and Western Africa	granulomatous / purulent dermatitis	GER, 2009*^A^	this study
10RB9208	African bull frog^2^ (wc)	Southeast, Central and Western Africa	granulomatous / purulent dermatitis	GER, 2009*^A^	this study
10RB9209	African bull frog^2^ (wc)	Southeast, Central and Western Africa	granulomatous / purulent dermatitis	GER, 2009*^A^	this study
10RB9210	African bull frog^2^ (wc)	Southeast, Central and Western Africa	granulomatous / purulent dermatitis	GER, 2009*^A^	this study
10RB9211	African bull frog^2^ (wc)	Southeast, Central and Western Africa	granulomatous / purulent dermatitis	GER, 2009*^A^	this study
10RB9212	African bull frog^2^ (wc)	Southeast, Central and Western Africa	granulomatous / purulent dermatitis	GER, 2009*^A^	this study
10RB9213	African bull frog^2^ (wc)	Southeast, Central and Western Africa	granulomatous / purulent dermatitis	GER, 2009*^A^	this study
10RB9214	African bull frog^2^ (wc)	Southeast, Central and Western Africa	granulomatous / purulent dermatitis	GER, 2009*^A^	this study
10RB9215	African bull frog^2^ (wc)	Southeast, Central and Western Africa	granulomatous / purulent dermatitis	GER, 2009*^A^	(7)
10RB9216	African bull frog^2^ (wc)	Southeast, Central and Western Africa	granulomatous / purulent dermatitis	GER, 2009*^A^	this study
10RB9217	African bull frog^2^ (wc)	Southeast, Central and Western Africa	granulomatous / purulent dermatitis	GER, 2009*^A^	this study
10-7-D-02627	Red-eyed tree frog^3^ (cb)	Central America	hind leg abscess	GER, 2010*	this study
152	Big-eyed tree frog^4^ (wc)	Tanzania	subcutaneous abscesses	GER, 2012^§^	(8)
141006639	Amazonian milk frog^5^ (cb)	South America	solid granuloma between heart / liver	GER, 2014*	this study
141006992	Amazonian milk frog^5^ (cb)	South America	no macroscopic lesions	GER, 2014*^†^	this study
UK8/14	Whites tree frog^6^ (cb)	Australia, New Guinea	fluid-filled skin lesions	UK, 2014^‡^	(9)
151–1	Amazonian milk frog^5^ (cb)	South America	swollen paravertebral ganglia	GER, 2015^B^	this study
214–1	Tomato frog^7^ (cb)	Madagascar	unilateral discolored kidney	GER, 2015^B^	this study
236–1	Tomato frog^7^ (cb)	Madagascar	focal white liver spot	GER, 2015^B^	this study
276–1	Tomato frog^7^ (cb)	Madagascar	reddened lung, unilateral enlarged kidney, mottled liver, enlarged spleen	GER, 2015^B^	this study
276–5	Tomato frog^7^ (cb)	Madagascar	reddened lung, unilateral enlarged kidney, mottled liver, enlarged spleen	GER, 2015^B^	this study

^1^: *Chaunus marinus*

^2^: *Pyxicephalus edulis*

^3^: *Agalychnis callidryas*

^4^: *Leptopelis vermiculatus*

^5^: *Trachycephalus resinifictrix*

^6^: *Litoria caerulea*

^7^: *Dyscophus antongilii*

AUS: Australia; GER: Germany; UK: United Kingdom

*: imported from Tanzania

^A^: zoo A

^B^: zoo B

^§^: purchased from a pet shop

^‡^: tropical animal collection

^†^: cage mate of 141006639

More detailed information about pathological findings among the various frog species can be retrieved from a recent publication by Muehldorfer et al. [12].

### *RecA* gene analysis

The *recA* genes of all 36 isolates from amphibians were amplified, sequenced and compared phylogenetically as described previously [13]. Briefly, the primer pair *recA*-BrucOchro-f (5`-atgtctcaaaattcattgcgac-3`) / *recA*-BrucOchro-r (5`-AGCATCTTCTTCCGGTCCGC-3´) was used to amplify a 1065 bp *recA* gene fragment. Trimmed partial sequences (628 bp) were aligned using MUSCLE implemented in MEGA v. 6 [14]. Phylogenetic reconstructions were performed using the neighbor joining (Kimura 2-parameter substitution model) and maximum likelihood (Jukes-Cantor) methods of MEGA with 500 repetitions. The type strains of *O*. *anthropi* and *O*. *intermedium* served as outgroup. The *recA* gene sequences from strains *B*. *inopinata* BO1, BO2, 83–13, and NF 2653 were extracted from the genome sequences available at (http://www.broadinstitute.org/) or (https://www.patricbrc.org).

### Genome sequencing

The genome sequence of strain (10RB9215), isolated from an African bullfrog, was determined using the PacBio (Pacific Biosiences, Menio Park, USA) sequencing platform. Briefly, DNA was extracted using the Qiagen spin column kit (Qiagen, Hilden, Germany). Genome sequencing was carried out by a sequencing company (GATC, Konstanz, Germany). Assembly of the PacBio generated reads (1 SMRT cell) was done using the freely available SMRT Analysis software (v. 2.3.0) (http://www.pacb.com/devnet/), and the HGAP3 algorithm with a read length minimum of 2500 bp. Genes that have no orthologues (singletons) in classical *Brucella* species were calculated using the singletons option of the EDGAR platform (available at https://edgar.computational.bio.uni-giessen.de) and an in-house *Brucella* genome database consisting of all known classical *Brucella* species. Identified singletons were further compared to the RefSeq database using the BLAST+ implementation of BLASTP with an initial evalue cutoff of 1e-10. Results were filtered for the best two hits of every query and the annotation and source organism for these best hits were extracted from the database.

### Phylogenetic reconstruction

Core genome-based phylogenetic trees were constructed as described in Blom et al. [15] using the EDGAR platform. Briefly, the core genome with *B*. *melitensis* 16M^T^ as the reference genome and the type strains and biovars of various *Brucella* species was calculated using the implemented function of EDGAR. The core genome consisted of 2466 coding sequences (CDS) per genome. Multiple alignments of the nucleotide coding sequences or their translated products were created for all core genes using MUSCLE [16]. The gene set alignments were concatenated to one large multiple alignment. Finally, phylogenetic trees (nucleic acid- and protein based) were generated using the F84 (DNA) or Kimura (AA) distance matrix and the neighbour joining method with 200 repetitions as implemented in PHYLIP [17]. Genome sequences used in this project were retrieved from either (http://www.ebi.ac.uk/genomes/) or (https://www.patricbrc.org).

## Results

### Genetic diversity

Thirty-six *Brucella* isolates collected from seven different exotic frog species were investigated by *recA* gene analysis to obtain a better understanding of their genetic diversity and their phylogenetic relationships to classical *Brucella* species infecting humans. Although *recA* provides no genetic resolution among the classical *Brucella* species, it is a powerful phylogenetic marker to delineate ‘atypical’ *Brucella* species and provides highly discriminatory resolution among the various *Ochrobactrum* species, the closest phylogenetic neighbors of *Brucella* [13].

**Fig 1:**
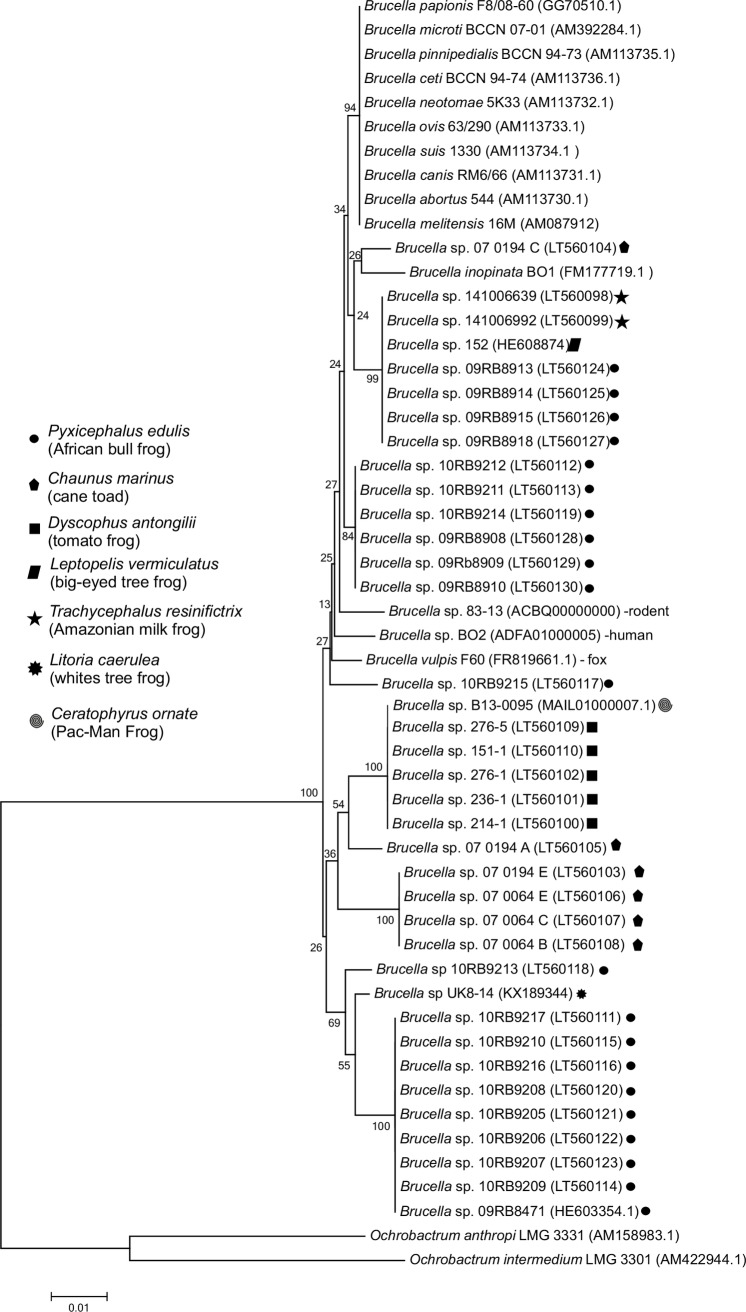
*Phylogenetic tree* from maximum likelihood analysis of the *recA* gene alignment of *Brucella* isolates from exotic frogs including classical and ‘atypical’ *Brucella* species. The tree was calculated with 100 bootstrap repetitions. *Ochrobactrum* served as outgroup. Bar: 0.08 substitutions per site. Accession numbers are given in brackets.

*RecA* gene cluster analysis revealed an unexpectedly high genetic heterogeneity among the various frog strains, not observed in classical *Brucella* species (Fig 1). Notably, isolates from the same frog species and origin were allocated to different *recA*- clusters whereas other clusters with identical *recA* gene sequences were formed by various frog species of different geographical origin. The 21 isolates from African bullfrogs formed three major clusters, consisting of nine, six, and four isolates, respectively. Two of the 21 strains (10RB9213 and 10RB9215) formed single lineages. The UK isolate from the white tree frog represented a singleton isolate most closely related to the largest of these groups. In contrast, the classical *Brucella* species and also *B*. *microti* and *B*. *papionis* were indistinguishable by means of their *recA* gene sequences. Notably, *B*. *inopinata* BO1 and unclassified strain BO2, both isolated from human infections, clustered within the frog isolates, indicating a close relationship and a possible source of infection. Four of the Australian isolates formed a novel clade. One strain (07/0194C) from a cane toad grouped together with *B*. *inopinata* BO1. The Australian rodent isolates 83–13 and NF 2653 had identical *recA* sequences and grouped separately within the frog isolates. Strain B13-0095, recently isolated from a Pac-Man frog (origin South America) clustered together with tomato frogs from Madagascar and was indistinguishable by means of its *recA* sequence.

### Genome sequencing of strain 10RB9215 and annotation

One African bull frog strain (10RB9215) isolated from a granulomatous / purulent skin lesion was selected for whole genome sequencing and compared with available genomes of classical *Brucella* species in order to shed light on the evolutionary background of amphibian brucellae. De novo assembly of the PacBio reads resulted in a high quality genome, consisting of two closed contigs (one for each chromosome) with a total size of 3,562813 bp and an average coverage of 120 x. The sizes of the chromosomes were 2.256.786 bp and 1.306.027 bp, respectively. The GC content was 57.2%. Annotation using RAST (http://rast.nmpdr.org/) revealed a total of 3279 coding sequences (CDS) and 67 RNAs.

### Core-genome based phylogenetic reconstruction

**Fig 2:**
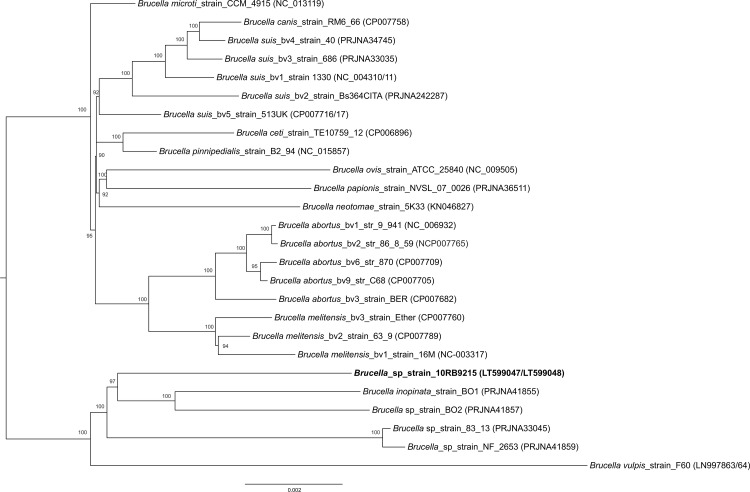
Core-genome-based phylogenetic neighbor-joining tree with 200 repetitions. Bar: 0.002 substitutions per site. Isolate 10RB9215 is indicated in bold letters. Accession numbers are given in brackets.

The genome-based phylogenetic reconstruction of strain 10RB9215 and other known *Brucella* species and their biovars revealed two major clusters (Fig 2), formed by the classical *Brucella* species together with *B*. *microti* and *B*. *papionis* and the distant brucellae, consisting of frog isolate 10RB9215, *B*. *inopinata* BO1, *B*. *vulpis* F60, strain BO2, and the Australian rodent isolates 83–13 and NF 2653. Strain 10RB9215 formed a separate lineage and was most closely related to *B*. *inopinata* and the *B*. *inopinata* related isolate BO2.

### Genes present in 10RB9215 but absent from classical *Brucella* species

Singleton analysis of strain 10RB9215 resulted in a total of 338 genes (10.3% of the entire coding sequences) absent from all classical *Brucella* species (S1 Table). Of the 338 singletons, 277 CDS had a BLAST hit against the reference database. Most of the hits with average similarities of 85–100% were detected with other atypical brucellae (*B*. *inopinata* and strain BO2) and *Ochrobactrum*, the closest genetic relative within the family *Brucellaceae*, a genus largely consisting of environmental bacteria occasionally infecting humans. Other hits were specific for a wide range of soil living or facultative pathogenic *Alphaproteobacteria*, like *Rhizobium*, *Agrobacterium*, *Mesorhizobium*, *Paracoccus* and *Bartonella*. The majority of genes with a BLAST hit (27.7%) encoded for additional phages and prophages, followed by genes for metabolism/ABC transporters (18.6%), carbohydrate metabolism (10.8%), and iron acquisition (5.4%). Several novel transposases, integrases and a DNA-modification system were identified. A list of all singletons together with the coding sequences and possible function is provided as supplementary material (S1 Table).

Many of the additional genes were organized in clusters (S1 Table), either flanked by mobile genetic elements or t-RNAs in combination with bacteriophages (not shown), suggesting extensive horizontal gene transfer. One of these clusters, encoding an ectoine uptake system is located on a 12,7 kb fragment on chromosome 1 (peg 1652–1661). The cluster, which is absent from all classical *Brucella* species consists of nine genes and shows high sequence similarity (av. 94%), to the ectoine system found in *Ochrobactrum* (S1 Table).

Ectoine (1,4,5,6-tetrahydro-2-methyl-4-pyrimidinecarboxylic acid) is a natural compound found in a wide range of Gram-negative and Gram-positive bacteria. It acts as an osmolyte and confers resistance towards salt and temperature [18]. Only recently a similar ectoine system has been described in an atypical Brucella isolate from a Pac-Man frog (*Ceratophrys ornata*). In the latter case ectoine could be used as a sole carbon source.

Another feature shared among strain 10RB9215 and members of the atypical *Brucella* clade (*B*. *inopinata*, BO2 and the Pac-Man isolate B13-0095) is the presence of a L-Rhamnose utilization gene cluster, a deoxy-hexose sugar commonly found in nature. Like in the other members of atypical brucellae the L-Rhamnose gene cluster of isolate 10RB9215 is located in close proximity to a flagellum gene cluster (not shown). The entire *virB* operon (B1-B11) is found on chromosome 2 (peg 1036–1046) of strain 10RB9215. In classical Brucella species this operon is important for virulence and is essential for the intracellular survival and multiplication within macrophages.

## Discussion

Until recently the genus *Brucella* was considered to represent a genetically homogeneous and clonal group of bacteria intricately associated with mammalian hosts. The data presented here illustrate a much more complex ecological situation with extant *Brucella* species representing specialized ecotypes with yet undetermined pathogenic potential for human and animal health. They form a background of an apparently genetically and geographically disparate population of ‘atypical’ *Brucella*. In contrast to known classical *Brucella* species, recently reported brucellae from exotic frogs are genetically highly diverse and might represent several new *Brucella* species. The presence of additional genetic material from various other soil-living bacteria suggests extensive horizontal gene transfer and indicates a different ecology compared to classical *Brucella* species which to date have been considered largely clonal [19]. It appears that the amphibian isolates may represent a link between free living soil saprophytes and the pathogenic *Brucella* with a preferred intracellular habitat, and further study of these organisms may help understanding the crucial steps in the evolution of virulence in the latter group. The close relationship of amphibian isolates with ‘atypical’ *Brucella* isolates, previously been associated with severe human disease, suggests that these organisms may themselves have pathogenic potential that merits investigation. Apparently, amphibian brucellae are capable of causing disease in different anuran species ranging from localized manifestations to generalized organ infections [7–12] (this study). This indicates that they are at least facultative pathogens of veterinary importance in cold-blooded vertebrates. Further, the world-wide distribution in frogs suggests that amphibians are not only occasionally infected by ‘atypical’ brucellae, but may represent a yet undiscovered and ecologically significant natural host for this group.

## Supporting Information

S1 Table Results of a BLAST analysis of singletons present in strain 10RB9215 but absent from classical *Brucella* species. A maximum of two BLAST hits are given. C1 = chromosome 1; c2 = chromosome 2.
(XLSX)
